# Biochemical and Functional Characterization of GALT8, an *Arabidopsis* GT31 β-(1,3)-Galactosyltransferase That Influences Seedling Development

**DOI:** 10.3389/fpls.2021.678564

**Published:** 2021-05-25

**Authors:** Joan Oñate Narciso, Wei Zeng, Kris Ford, Edwin R. Lampugnani, John Humphries, Ingvild Austarheim, Allison van de Meene, Antony Bacic, Monika S. Doblin

**Affiliations:** ^1^ARC Centre of Excellence on Plant Cell Walls, School of BioSciences, The University of Melbourne, Melbourne, VIC, Australia; ^2^Sino-Australia Plant Cell Wall Research Centre, State Key Laboratory of Subtropical Silviculture, School of Forestry and Biotechnology, Zhejiang A & F University, Hangzhou, China

**Keywords:** plant cell wall, seedling development, galactosyltransferase, arabinogalactan-proteins, *Arabidopsis thaliana*, *GALT8*, *KNS4/UPEX1*

## Abstract

Arabinogalactan-proteins (AGPs) are members of the hydroxyproline-rich glycoprotein (HRGP) superfamily, a group of highly diverse proteoglycans that are present in the cell wall, plasma membrane as well as secretions of almost all plants, with important roles in many developmental processes. The role of GALT8 (At1g22015), a Glycosyltransferase-31 (GT31) family member of the Carbohydrate-Active Enzyme database (CAZy), was examined by biochemical characterization and phenotypic analysis of a *galt8* mutant line. To characterize its catalytic function, GALT8 was heterologously expressed in tobacco leaves and its enzymatic activity tested. GALT8 was shown to be a β-(1,3)-galactosyltransferase (GalT) that catalyzes the synthesis of a β-(1,3)-galactan, similar to the *in vitro* activity of KNS4/UPEX1 (At1g33430), a homologous GT31 member previously shown to have this activity. Liquid chromatography-tandem mass spectrometry (LC-MS/MS) confirmed the products were of 2-6 degree of polymerisation (DP). Previous reporter studies showed that *GALT8* is expressed in the central and synergid cells, from whence the micropylar endosperm originates after the fertilization of the central cell of the ovule. Homozygous mutants have multiple seedling phenotypes including significantly shorter hypocotyls and smaller leaf area compared to wild type (WT) that are attributable to defects in female gametophyte and/or endosperm development. *KNS4/UPEX1* was shown to partially complement the *galt8* mutant phenotypes in genetic complementation assays suggesting a similar but not identical role compared to *GALT8* in β-(1,3)-galactan biosynthesis. Taken together, these data add further evidence of the important roles GT31 β-(1,3)-GalTs play in elaborating type II AGs that decorate AGPs and pectins, thereby imparting functional consequences on plant growth and development.

## Introduction

Arabinogalactan-proteins (AGPs), found exclusively in plants and in virtually all organs/tissues, are the most highly glycosylated members of the hydroxyproline-rich glycoprotein (HRGP) superfamily (Nothnagel, 1997; Ellis et al., 2010; Nguema-Ona et al., 2012; Tan et al., 2012). AGPs are proposed to have a wide range of physiological and developmental functions related to vegetative processes and sexual reproduction (Ellis et al., 2010). In general, AGP protein backbones contain clustered, non-contiguous proline (Pro) residues arranged in repetitive motifs such as Xaa-Pro-Xaa-Pro, and Xaa-Pro-Pro, where Xaa can be any amino acid, but is most often either Ser, Thr, Val, or Ala (Nothnagel, 1997). Indeed, a recent motif and amino acid bias (MAAB) bioinformatics pipeline reported using the [ASVTG]P, [ASVTG]PP, and [AVTG]PP motifs to identify AGPs within the Viridiplantae (Johnson et al., 2017a, b). These types of Pro residues are usually hydroxylated forming hydroxyproline (Hyp) and then glycosylated by type II arabino-3,6-galactan (AG) chains although short mono-/oligo-arabinosides can also be present; a pattern of glycosylation that is defined by the Hyp-contiguity hypothesis (Kieliszewski and Lamport, 1994; Lamport et al., 2011). AGPs are also distinguished from other members of the HRGP superfamily by their ability to bind selectively to, and be precipitated by Yariv reagents (Tan et al., 2012; Kitazawa et al., 2013).

Despite significant progress in AGP research in recent years, the contribution of AG oligo-/poly-saccharides to AGP function remains largely inferred (Tan et al., 2012). Characterization of genetic mutants of glycosyltransferases (GTs) responsible for AGP glycosylation has provided clues to the biological function(s) for the AG glycans but the effects are often pleiotropic and therefore difficult to ascribe to a single AGP protein backbone. For recent reviews on characterized AGP GTs, we refer the reader to Knoch et al. (2014); Showalter and Basu (2016), Ma et al. (2018); Silva et al. (2020).

Within each AG moiety of AGPs, there are 30 to 120 sugar residues (Ellis et al., 2010). Although AGP glycan structures isolated from various plants differ from each other, the type II AGs generally contain β-(1,3)-linked Gal backbones with β-(1,6)-linked Gal side-chains decorated with Ara, GlcA, Rha, Fuc, and/or Xyl residues (Ellis et al., 2010; Tan et al., 2012). The heterogeneity of AGs and the fact that AGPs are often purified as mixtures of related structural entities contribute to the challenges that arise from characterizing the fine structures of the carbohydrate moiety of AGPs. The composition and structure of AGs varies within a species, even on the same peptide backbone and may be tissue-specific and developmentally regulated (Tsumuraya et al., 1988; Estevez et al., 2006). It is likely that multiple GTs with partially overlapping expression patterns work together to generate the observed complexity of AGP glycans, possibly working as biosynthetic complexes as observed for many wall polysaccharides such as cellulose (Doblin et al., 2002; Lei et al., 2012), xyloglucan (Chou et al., 2012), xylan (Zeng et al., 2016), and pectin (Atmodjo et al., 2011; Oikawa et al., 2013).

Identifying the enzymes involved in the synthesis of the sugar linkages in AGPs is not a trivial task. Because there are many different types of linkages in AGP glycan moieties, and there are many possible enzyme families that can be involved, it is apparent that narrowing down the number of enzyme candidates is a crucial step in elucidating the pathways in type II AG biosynthesis. To this end, Qu et al. (2008) identified, through a bioinformatics approach, a group of 20 putative β-(1,3)-galactosyltransferases (GalTs) in *Arabidopsis thaliana* that were plausibly responsible for the β-(1,3)-Gal linkages in AGPs. These enzymes are classified within the glycosyltransferase (GT) 31 family (Clade 7 and 10 members, Supplementary Figure 1) in the Carbohydrate-Active Enzymes (CAZy) database (^1^ Lombard et al., 2013) because of the presence of PFAM domain PF01762 and several motifs in their protein sequences that are found in biochemically characterized β-(1,3)-GTs in mammalian systems (Egelund et al., 2010). Toward the N-terminus of the GT31 enzymes is a conserved hydrophobic transmembrane domain (TMD) typical of a type 2 membrane protein, followed in the case of Clade 7 members by a galactoside-binding lectin (galectin) domain (Supplementary Figure 1). This galectin domain could be responsible for the addition of the first or subsequent Gal to the polypeptide backbone of AGPs based on the activity of human galectin domain proteins (Egelund et al., 2010). This domain is followed by the six major conserved motifs I-VI, three of which (RxxxRxT/SW (II)), DXD (IV), E/DDV and GxW/C (VI) are critical for catalysis (Munro and Freeman, 2000; Griffitts et al., 2001; Hellberg et al., 2002; Malissard et al., 2002; Qu et al., 2008; Egelund et al., 2010).

Interestingly, both galectin (Clade 7, sub-clades V and VI (Qu et al., 2008)) and non-galectin-containing (Clade 10, sub-clade III, Supplementary Figure 1) GT31 members have been demonstrated to have *O*-Hyp GalT activity, adding the first Gal sugar to Hyp residues within the AGP protein backbone (Basu et al., 2013; Ogawa-Ohnishi and Matsubayashi, 2015; Basu et al., 2015a, b). Qu et al. (2008) showed that an *Arabidopsis* membrane fraction containing Clade 10 member *At1g77810* (sub-clade II, Supplementary Figure 1) has β-(1,3)-GalT activity using UDP-Gal as the donor substrate and Gal-(1,3)-β-Gal-O-Me as acceptor. More recently, Suzuki et al. (2017) published the first detailed biochemical analysis of a β-(1,3)-galactan synthase, KNS4/UPEX1 (At1g33430) from Clade 10 (sub-clade II, Qu et al., 2008). Given that this enzyme can transfer multiple Gal residues from UDP-Gal to Gal-NBD, a synthetic acceptor that mimics the GalT acceptor in the biosynthesis AG oligosaccharides, it is proposed to be responsible for the synthesis of type II AG glycan backbones present on either AGPs or the pectic polysaccharide rhamnogalacturonan I (RG-I), or both. The defective exine phenotype of *kns4/upex1* mutants is related to an abnormality of the primexine matrix laid on the surface of developing microspores and is associated with diminished levels of AGPs. Furthermore, *kns4/upex1* mutants exhibit reduced fertility as indicated by shorter fruit lengths and lower seed set compared to the WT, confirming that *KNS4/UPEX1* is critical for pollen viability and development.

We are interested in GALT8 (At1g22015), also called DD46 (Portereiko et al., 2006), for two reasons: First, GALT8 is the only uncharacterized *Arabidopsis* member of sub-clade II of Clade 10 (Supplementary Figure 1). KNS4/UPEX1, At1g77810, and GALT31A, which was reported to possess β-(1,6)-GalT activity (Geshi et al., 2013), are also members of this sub-clade. Thus, the variation in observed enzymatic activity makes it difficult to predict the catalytic function of GALT8 and its role *in planta*. Second, promoter-reporter (GFP and GUS) constructs demonstrated that *GALT8* is expressed in the female gametophyte prior to fertilization and is important in embryo sac development (Wu et al., 2010; Gille et al., 2013). This pattern contrasts with *KNS4/UPEX1*, which is primarily expressed in, and influences development of, the male gametophyte. This suggests that *galt8* mutants may display a phenotype, a valuable resource in dissecting AGP function, and which is distinct from that displayed by *kns4/upex1* mutants.

In this study, we show using a combination of enzymatic digestion, RP-HPLC and MS-based approaches that GALT8 is a β-(1,3)-GalT capable of catalyzing the addition of up to five β-(1,3)-linked Gal residues onto a fluorescently tagged β-Gal acceptor. We also show by semi-quantitative RT-PCR that *GALT8* is expressed in anther, pistil and silique, confirming previous gene expression data (Portereiko et al., 2006; Leroy et al., 2007). *Galt8* mutant seedlings have observable phenotypes that are consistent with *GALT8* functioning in seedling growth and development through potential perturbation of the micropylar endosperm. Genetic complementation experiments were also performed to test whether *GALT8* and *KNS4/UPEX1* are functionally equivalent. We demonstrate that expression of *KNS4/UPEX1* in the *galt8* background under the native *GALT8* promoter partially complemented the *galt8* phenotypes, suggesting GALT8 and KNS4/UPEX1 have both overlapping and unique functions *in planta*. Together, these data provide evidence that, similar to KNS4/UPEX1, GALT8 is an *Arabidopsis* β-(1,3)-galactosyltransferase associated with type II AG glycan biosynthesis and is important in normal seedling growth and development.

## Materials and Methods

### Plant Material

*Arabidopsis thaliana* wild type (WT) Columbia-0 (Col-0), *kns4-2* (SALK_091466 line) and *galt8* (SALK_208637C line) mutant seeds were obtained from the Nottingham Arabidopsis Stock Centre (NASC, University of Nottingham, United Kingdom). Plants were sown on a 3:1 mix of soil (Debco seed raising mix, Tyabb, VIC, Australia) and Perlite (Exfoliators, Australia), stratified at 4°C for 3 days, then transferred to a controlled-environment growth chamber (Thermoline, Australia) at 21°C under continuous light. The mutants were compared to the WT Col-0 ecotype. *Nicotiana benthamiana* plants were grown in soil in a glasshouse supplemented with continuous cool white light at 20 to 26°C for 4 to 5 weeks. Phenotyping of *galt8* and WT Col-0 plants, as well as plant crosses were performed following the protocol from Weigel and Glazebrook (2002).

### Molecular Biology

Individual 3-week-old seedlings were genotyped by PCR screening of genomic DNA prepared using the ISOLATE II Plant DNA Kit (Bioline, Australia) following the manufacturer’s instructions. A typical PCR contained 1 μl of gDNA (at least 100 ng), 0.2 mM forward and reverse primers spanning or within the T-DNA insertion (Supplementary Table 1) and 5 U MyTaq^TM^ DNA Polymerase in 1× MyTaq^TM^ reaction buffer (Bioline, Australia). Conditions for PCR amplification were as follows: 94°C for 45 s; 55°C for 45 s; 72°C for 1 min for 30 cycles.

For *GALT8* RT-PCR analysis, RNA was isolated from various *Arabidopsis* WT Col-0 and *galt8* tissues, including floral buds used as a template source for coding sequence (CDS) cloning, and first strand cDNA synthesis and PCR amplifications were carried out as previously described (Lampugnani et al., 2013) (see Supplementary Table 1 for primer details). For quantitative RT-PCR analysis of *galt8* complementation lines, RNA was isolated from hypocotyls of light-grown 6-day-old seedlings using the Spectrum Plant Total RNA Kit (Merck, Australia) according to manufacturer’s instructions. DNase treatment was carried out using the DNase I kit (Invitrogen #18068-015) and cDNA was synthesized from 1 μg of RNA using the SuperScript III Reverse Transcriptase kit (Invitrogen). cDNA was diluted 1:10 and used as template in qRT-PCR reactions performed with the QuantStudio 5 system (Applied Biosystems) using the SensiMix SYBR No-ROX kit (Bioline). Reaction conditions were as follows: 95°C initial hold for 10 min, then 40 cycles of 95°C for 15 s and 60°C for 60 s. Relative expression of *GALT8* and *KSN4/UPEX1* were determined using the Comparative Ct (ΔΔCt) method, with *GLYCERALDEHYDE-3-PHOSPHATE DEHYDROGENASE* (*GAPDH*) as a reference gene. Primers used for amplification of *GALT8*, *KNS4/UPEX1* and *GAPDH* are listed in Supplementary Table 1.

To generate pro35S:GALT8, pro35S:GALT8(CΔ106), and pro35S:GALT8(CΔ168) expression constructs, the *GALT8* coding sequence (CDS) was amplified from floral bud cDNA using primer pairs GALT8(1)-F & GALT8(2)-R, GALT8(3)-F & GALT8(4)-R and GALT8(3)-F & GALT8(5)-R separately (see Supplementary Figure 2A). The PCR products were cloned directly into the binary vector pFUERTE containing the CaMV35S promoter and the 3′ OCS terminator sequence (Lampugnani et al., 2016), using New England Biolabs HiFi DNA assembly reagents and the protocol specified by the manufacturer. To generate pro35S:GALT8(NDN) we utilized the resulting pro35S:GALT8 plasmid as a template for PCRs using primers GALT8(3)-F & GALT8(6)-R and separately GALT8(7)-F & GALT8(8)-R. Both fragments were simultaneously cloned into pFUERTE as described above. Primer sequences used in these clonings are provided in Supplementary Table 1. The pro35S:VENUS construct has been previously described (Suzuki et al., 2017). All constructs were introduced, together with pSOUP, into *Agrobacterium tumefaciens* strain AGL1 via electroporation.

To generate GALT8/KNS4 complementation constructs, promoter regions of *GALT8* and *KNS4/UPEX1* were individually amplified from *Arabidopsis* genomic DNA using primer pairs GALT8PromF_*Eco*RI & GALT8PromR_*Sal*I and KNS4PromF_*Eco*RI & KNS4PromR_*Sal*I, respectively, incorporating *Eco*RI and *Sal*I restriction sites (see Supplementary Figure 2B). Similarly, *GALT8* and *KNS4/UPEX1* CDS were amplified from *Arabidopsis* floral bud cDNA by primer pairs GALT8F_*Sal*I & GALT8R_*Nco*I and KNS4F_*Sal*I & KNS4R_*Nco*I, respectively, with flanking restriction sites *Sal*I and *Nco*I. Purified fragments were digested with the appropriate restriction enzyme, and combinations of promoter and coding region were simultaneously ligated into the multiple cloning site of pCAMBIA0380 digested with *Eco*RI & *Nco*I using T4 DNA ligase (Promega) following the manufacturer’s instructions. The common *Sal*I site enabled fusion of the promoter and CDS regions. The pCAMBIA0380 vector possesses the neomycin phosphotransferase II (NPTII) gene for plant selection on kanamycin as does pGWB-KNS4p:KNS4, the vector used previously to genetically complement the *kns4-1* mutant (Suzuki et al., 2017). This construct was used as a positive control [proKNS4:KNS4 (2)] and contains 2006 bp of promoter sequence. The promoter version cloned into pCAMBIA0380 [proKNS4:KNS4] is 1327 bp in length and was designed to contain the entire upstream intergenic sequence, the design principle also applied to amplify and clone the GALT8 promoter (853 bp). All verified constructs were introduced into *Agrobacterium tumefaciens* strain AGL1 via electroporation. Primer sequences used for complementation constructs are provided in Supplementary Table 2.

### Functional Analysis of GALT8 Enzyme Activity

The methods for transient expression of GalT in *Nicotiana benthamiana*, microsomal membrane (MM) extraction, and GalT biochemical activity assay were conducted as previously described by Suzuki et al. (2017), using 5 μM fluorescent acceptor β-Gal-NBD [NBD = 7-nitro-2,1,3-benzoxadiazole; (McGill and Williams, 2009)] kindly provided by Professor Spencer Williams (Department of Chemistry, Bio21 Institute, The University of Melbourne, Australia), and 0.4 mM UDP-sugar (Sigma-Aldrich) as donor. The total reaction volume used for enzyme assays was 50 μL containing 100 μg detergent-permeabilized MM as determined by a BCA protein assay (Pierce) using BSA as standard. When assaying GALT8 variants, MM protein concentrations were normalized for GALT8 content as determined by Western blotting using a polyclonal GALT8 antibody as probe. GALT8 band intensities were quantified and compared to an arbitrarily selected MM sample in which GALT8 was detected. MM volumes of other GALT8-containing samples were adjusted such that approximately equal amounts of GALT8 protein was assayed in enzyme reactions. Western blotting was performed as described previously (Wilson et al., 2015) using a 1:2000 dilution of anti-GALT8 primary antibody raised against peptide YAHEKKKSQDNDVMC (C-terminal Cys residue included for conjugation to carrier protein) using GenScript’s Custom Rabbit Antibody Services (Piscataway, NJ, United States).

For the analysis of assay products, ice-cold acetone was added to the enzyme assay (3:1 volume), and the mixture incubated on ice for 2 h. The reaction was then centrifuged at maximum speed at 4°C, the supernatant removed and dried down, re-suspended in 50 μL UHQ water, and analyzed by nano-LC-MS/MS. The sample was loaded onto a 300 μm × 5 mm Zorbax 300SB-C18 (Agilent Technologies, Palo Alto, CA, United States) reversed-phase (RP) pre-column attached to a Shimadzu Prominence nano-LC system (Shimadzu Corporation, Kyoto, Japan). The pre-column was washed with 0.1% (v/v) formic acid in 5% (v/v) acetonitrile for 15 min before placing in-line with a 75 μm i.d. ×150 mm Zorbax 300SB-C18 (Agilent Technologies, Palo Alto, CA, United States) RP column. Products were eluted using a gradient of 5% to 80% (v/v) acetonitrile in 0.1% (v/v) formic acid over 60 min, at a flow rate of 0.2 μL/min and analyzed via electrospray ionization (ESI) on a QSTAR Elite hybrid Q-TOF MS (Applied Biosystems/MDS Sciex, Foster City, CA, United States) operated in the positive ion mode. Products between 300 Da and 1,500 Da, with a charge state of either +1 or +2 were automatically selected for MS/MS with a CE of 50. The molecular ion peaks of the different oligosaccharides in the enzyme assay, particularly [M + H]^+^ = 552.4, 714.5, 876.6, 1038.6, 1200.5, and 1362.5, corresponding to Gal_1_-NBD (C_23_H_33_N_7_O_9_), Gal_2_-NBD (C_29_H_43_N_7_O_14_), Gal_3_-NBD (C_35_H_53_N_7_O_19_), Gal_4_-NBD (C_41_H_63_N_7_O_24_), Gal_5_-NBD (C_47_H_73_N_7_O_29_), and Gal_6_-NBD (C_53_H_83_N_7_O_34_), respectively, were extracted from the full scan spectra.

### β-Glucosyl Yariv Radial-Gel Diffusion Assay

For detecting the presence of β-(1,3)-Gal linkages in the enzyme assay products, a β-glucosyl Yariv (β-Glc Yariv) radial-gel diffusion assay was performed following the method of van Holst and Clarke (1985), with slight modifications. Bulked enzyme assays with 1.5 mg of total MM proteins (adjusted for GALT8 depending on the relative quantity detected by Western blot), 1% (v/v) Triton X-100, 10 μM β-Gal-NBD, and 1 mM UDP-Gal (total volume for each enzyme assay was 250 μL) were loaded to the RP-HPLC column, and fractions corresponding to 4.0-4.5 min, 4.6-8.0 min, and 8.1-10.0 min were collected, lyophilized, and re-suspended in water. Samples were applied onto two agarose gels, one containing β-Glc Yariv dye, the other containing α-Gal Yariv dye as negative Yariv control, and allowed to react overnight. Gum arabic (0.1 mg/ml, 0.2 mg/ml, 0.5 mg/ml, and 1.0 mg/ml) (Sigma-Aldrich) was used as standard and positive control, and β-Gal-NBD and larch AG (Sigma-Aldrich) were used as negative controls.

## Results

### Analysis of GALT8 Biochemical Activity

**FIGURE 1:**
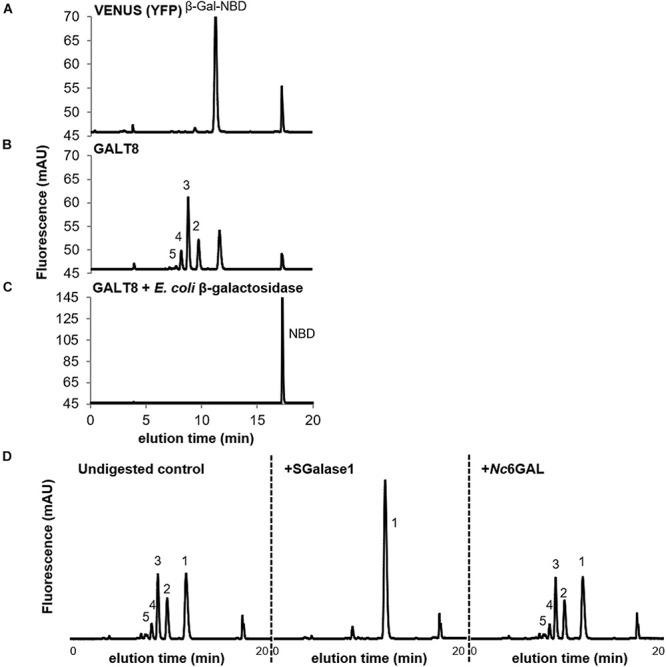
GalT activity of MM prepared from *N. benthamiana* leaves expressing VENUS or GALT8, and analysis of GALT8 reaction products. **(A)** VENUS, only β-Gal-NBD and NBD peaks are observed. **(B)** GALT8, products of DP 2-5 were detected. **(C)** Digestion of GALT8 assay products with commercially available *E. coli* β-galactosidase, showing digestion of peaks back to the NBD tag (R_t_ = ∼17.0 min). GalT products were fractionated by RP-HPLC and detected by a fluorescence detector. The numbers on the plot (**B**) indicate the degree of polymerisation (DP) of galacto-oligosaccharides. GalT activity was measured using UDP-Gal (0.4 mM) as donor and β-Gal-NBD (5 μM) as the acceptor. **(D)** Relative to undigested GALT8 assay products (left), SGalase1, an exo-β-(1,3)-galactanase, digested the enzyme products to Gal-NBD (center), whereas endo β-(1,6)-galactanase (*Nc*6GAL) did not (right), as shown by an unchanged profile. The numbers (1-5) on the plots indicate the DP of galacto-oligosaccharides.

To gain insight into the function of *GALT8* (At1g22015), the single uncharacterized sub-clade II member of the Arabidopsis GT31 family, we expressed the full-length *GALT8* coding sequence (CDS) under the CaMV35S promoter in *N. benthamiana* leaves. An identical binary vector construct carrying the YFP variant VENUS instead of the GALT8 CDS was used as a negative control to allow subtraction of the background endogenous enzyme activity. Microsomal membranes (MM) were isolated from leaves 4 days post-agroinfiltration and used in separate *in vitro* GalT enzyme assays each containing 0.4 mM UDP-Gal as the donor substrate and 5 μM of the synthetic AG oligosaccharide mimic β-Gal-NBD (McGill and Williams, 2009) as acceptor. The reaction products were subsequently fractionated by RP-HPLC and analyzed. No intrinsic GalT activity was observed in the VENUS sample with only the β-Gal-NBD acceptor being detected (Figure 1A). In contrast, four new peaks were observed in the GALT8 sample (Figure 1B), presumably representing the addition of up to four Gal residues to β-Gal-NBD. Other nucleotide sugar donors, UDP-Glc, UDP-Ara*p*, and UDP-Xyl, were also tested but the donor specificity was found to be restricted to UDP-Gal (Supplementary Figure 3); none of the other UDP-sugar donors tested resulted in the formation of any products using β-Gal-NBD as the acceptor.

To confirm that Gal was the incorporated monosaccharide, we tested the susceptibility of the assay products to digestion by a linkage non-specific *E. coli* β-galactosidase (CAZy GH2) (Shimoda et al., 2014). β-Galactosidase digestion of the GALT8 reaction products resulted in the breakdown of all products and the β-Gal-NBD acceptor to the free NBD tag, which eluted at ∼17.0 min (Figure 1C). This confirmed that the products made by GALT8 were β-D-Gal oligosaccharides.

To determine if the linkage present in the β-D-Gal oligosaccharide products was β-(1,3), the assay products were further characterized by digestion with a type II AG-specific hydrolase, SGalase1, an exo-β-(1,3)-D-galactanase (CAZy GH43) (Ling et al., 2012). SGalase1 hydrolyzed the GALT8 products to β-Gal-NBD (compare left and middle panels, Figure 1D). The enzyme products were also assessed for their susceptibility to an endo-β-(1,6)-galactanase, *Nc*6GAL (Takata et al., 2010). Following digestion with *Nc*6GAL, the resulting RP-HPLC profiles were unchanged from the undigested controls (compare left and right panels, Figure 1D), indicating the absence of β-(1,6)-Gal branches in these GalT products. Thus, the major products formed by GALT8 were chromatographically and enzymically deduced to be an oligomeric β-(1,3)-D-galacto-oligosaccharide series of degree of polymerisation (DP) 2-5.

**FIGURE 2:**
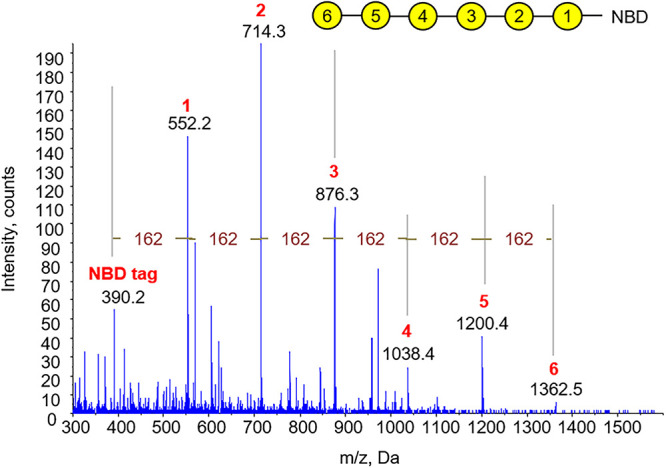
Characterization of GALT8 enzyme assay products by direct infusion ESI-MS and -MS^n^. MS spectrum generated in positive ion mode showing a hexose series of up to DP5 by direct infusion ESI-MS of acetone-treated GalT assay products, with a m/z Δ162 Da between peaks. The abundance of DP6 was too low to be further fragmented by MS^2^. Yellow circles represent Gal residues, numbers their relative position in the chain.

The composition of the GALT8 reaction products was further confirmed by direct infusion ESI-MS and -MS^n^ of the individual peaks collected upon RP-HPLC fractionation. The major MS ions were observed to be a hexose series due to the m/z ?162 Da between ions upon MS^2^ fragmentation (Figure 2). Ions corresponding to 1 - 5 Gal (hexose) unit additions to β-Gal-NBD were detected, as expected based on the RP-HPLC chromatogram.

### β-Glucosyl Yariv Radial-Gel Diffusion Assay of GALT8 Reaction Products

**FIGURE 3:**
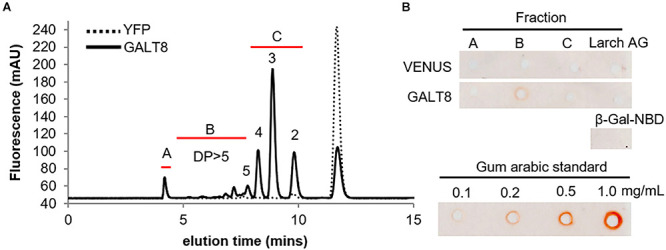
Characterization of GALT8 enzyme assay products by the β-glucosyl Yariv radial-gel diffusion assay. VENUS and GALT8 MM were extracted from *N. benthamiana* leaves transiently expressing the gene constructs. **(A)** Products from VENUS and GALT8 enzyme assays fractionated on RP-HPLC showing different collected fractions and their elution times (Fr. A: undefined higher DP; Fr. B: DP ≥ 5; and Fr. C: DP 2-4). **(B)** Fractions from RP-HPLC in a β-Glc Yariv radial-gel diffusion assay with gum arabic (GA) as positive control, and β-Gal-NBD and larch arabinogalactan (AG) as negative controls. Red halos indicate a positive reaction. Numbers above peaks indicate the number of Gal residues.

Reactivity toward β-Glc Yariv is a specific test for the presence of AGPs, binding preferentially to β-(1,3)-Gal backbones with DP > 5 (Kitazawa et al., 2013; Paulsen et al., 2014). Thus, to determine whether the enzyme assay products of GALT8 have a type II AG β-(1,3)-Gal structure, their reactivity toward β-Glc Yariv using the radial-gel diffusion assay (van Holst and Clarke, 1985) was tested. RP-HPLC fractions from bulked GALT8 enzyme assay products were collected (Figure 3A) and applied into wells in an agarose gel containing β-Glc Yariv reagent. Fraction B containing products with DP ≥ 5 reacted positively toward β-Glc Yariv, as indicated by a reddish-orange halo that is also observed with increasing intensity with the gum arabic standard (Figure 3B). Fraction A with undefined higher DP was negative, likely due to the limited quantity of such products. None of the fractions exhibited a halo in the control gel containing α-Gal Yariv that does not bind AGPs (data not shown). Similarly, neither Fraction C containing DP 2-4, β-Gal-NBD, nor larch AG reacted with either β-Glc- or α-Gal-Yariv reagents (Figure 3B). These results further support the enzymatic and LC-MS data indicating that the products of the GALT8 enzyme assay are β-(1,3)-galacto-oligosaccharides.

### Characterization of a *GALT8* T-DNA Insertion Mutant Line

**FIGURE 4:**
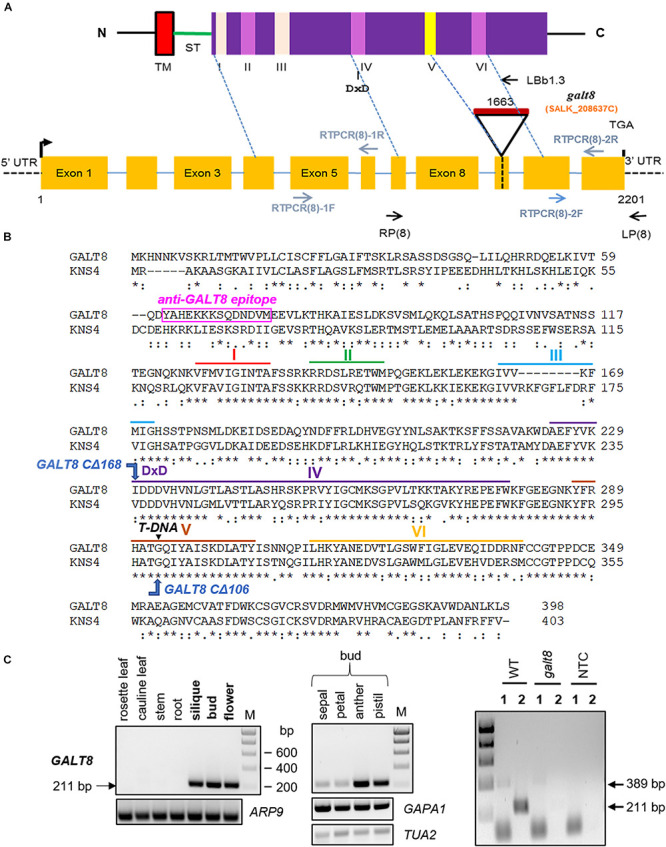
Molecular characterization of *GALT8* T-DNA insertion line. **(A)** Schematic diagram of the general protein domain structure and major motifs of GALT8 (**top**) and the *GALT8* locus (**bottom**). *Top:* N, N-terminus; C, C-terminus; TM, predicted trans-membrane region; ST, stem region; purple box, PFAM GalT (PF01762) domain; small pink and yellow boxes, six major conserved motifs I-VI. *Bottom:* yellow boxes, exons; blue lines, introns; dotted black lines, 5′ and 3′ UTRs; black triangle, position of T-DNA insertion; black hooked arrow, translation start site; black vertical line, stop codon. Primers used in genotyping and RT-PCR experiments are indicated with black and blue arrows, respectively. Numbers indicate nucleotides relative to the initiating AUG codon. **(B)** Protein alignment of GALT8 and KNS4/UPEX1 showing the sequences within motifs I-VI as shown in panel **(A)**. Motifs are as follows: I – a conserved hydrophobic region, II - RxxxRxT/SW, III - FxxG/A, IV - AxF/YxxK, DxD, LxYxG, PxR and PExW, V - YP/FxA/C, GxxYxxS and DxA, VI - E/DDV and GxW/C. Black triangle, T-DNA insertion site; pink rectangle, position and sequence of the anti-GALT8 peptide epitope. **(C)** Upper left and upper middle panels show the PCR products amplified from WT Col-0 cDNA using the RTPCR(8)-2F and -2R primer pair. Right panel shows knock-out homozygous *galt8* mutant line. The upper middle panel shows cDNA samples derived from floral organs at bud stages 10 to 12. Actin (*ARP9*), *GAPDH* (*GAPA1*), and tubulin (*TUA2*) were the housekeeping genes used as controls. Semi-quantitative-RT-PCR was performed on cDNA generated from *galt8* mutant lines using primer pairs that lie either before (1, RTPCR(8)-1F and -1R) or after (2, RTPCR(8)-2F and -2R) the T-DNA insertion site. Predicted PCR product sizes: (1) 389 bp, (2) 211 bp. M, Hyperladder I DNA marker; NTC, no template control.

To examine whether plants impaired in GALT8 function have a phenotype, seeds of a homozygous single-locus T-DNA insertion mutant line for *GALT8* (*galt8*, SALK_208637C) were obtained from the Nottingham Arabidopsis Stock Centre (NASC) and bulked for genotyping. The zygosity and position of the T-DNA insertion in individual plants were determined by PCR screening using two sets of primers flanking the T-DNA insertion site (Figure 4A). The T-DNA insertion in *galt8* was determined to be 1,663 bp downstream of the ATG codon, and 74 bp downstream of the position reported in the *Arabidopsis* T-DNA insertion database (SIGnAL^2^) (Figure 4A). The T-DNA insertion is just before the GxxYxxS sequence within conserved motif V in exon 9 (Figures 4A,B). Conserved motif V has a conserved glycine residue that is involved in donor binding (Taujale et al., 2020). It is therefore likely that the *galt8* transcript is non-functional due to the lack of conserved motif VI. The last conserved motif VI contains the E/DDV and GxW/C domains and is located near the C-terminus of the GT31. Together with the DXD motif, it is involved in catalytic functions of GT-A fold inverting mammalian GTs and other GT31 family members (Qu et al., 2008; Egelund et al., 2010; Taujale et al., 2020).

According to *Arabidopsis* microarray expression data (Arabidopsis eFP Browser 2.0^3^), *GALT8* is expressed in floral stages 9 to 12 when the floral organs rapidly elongate (Smyth et al., 1990), similar to *KNS4/UPEX1*, and appears to be specifically expressed in the micropylar endosperm in the pre-globular and globular stages post-fertilization (Supplementary Figure 4). To validate the microarray data, we analyzed by semi-quantitative RT-PCR the expression pattern of *GALT8* in a range of vegetative and floral tissues (Figure 4C, left and middle panels). Our results show that *GALT8* is expressed in floral buds (stages 10-12) as well as the mature flowers especially in the anther and pistil and in developing siliques. The expression of *GALT8* was subsequently tested in the *galt8* mutant line. The absence of a 211 bp band expected to be amplified by primers RT-PCR(8)-2F and RT-PCR(8)-2R in WT (WT, Col-0) indicates the loss of *GALT8* transcription after the T-DNA insertion site (Figure 4C, right panel), confirming the absence of the downstream protein sequence as predicted.

### Truncation and Site-Directed Mutagenesis of *GALT8*

**FIGURE 5:**
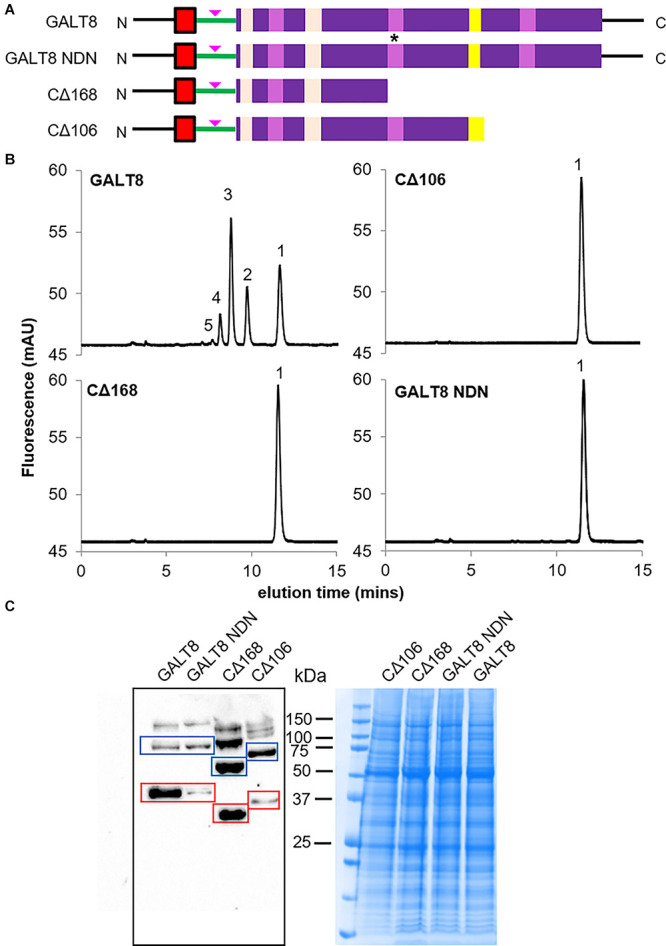
GalT activity and Western blot analysis of MM extracted from *N. benthamiana* leaves infiltrated with different truncated and mutated versions of *GALT8* gene constructs, and wild type *GALT8* as control. **(A)** Schematic diagrams of the *GALT8* expression constructs. *Top to bottom:* Wild type GALT8, GALT8 mutated in the DDD domain (motif IV) marked with an asterisk, D_231_D_232_D_233_ mutated to N_231_D_232_N_233_, NDN; GALT8 truncated before the DDD domain (CΔ168); GALT8 truncated at the T-DNA insertion site (a.a. 292 from N-terminal, CΔ106). Pink arrow indicates anti-GALT8 peptide epitope. **(B)** GALT activity assays using MM (100 μg protein) prepared from *N. benthamiana* leaves infiltrated with different *GALT8* gene constructs. **(C)** MM (100 μg protein) prepared from *N. benthamiana* leaves infiltrated with different *GALT8* gene constructs (**B**) analyzed via Western blotting under reducing conditions using anti-GALT8 antibody as probe. MW of wild type GALT8 and GALT8 NDN = 45.2 kDa; MW of GALT8 CΔ106 = 33.3 kDa; MW of GALT8 CΔ168 = 26.3 kDa (red boxes). Bands with twice the MW of the GALT8 monomer versions are indicated by blue boxes, and may suggest homodimers.

To examine the importance of a full-length GALT8 and the role of the DDD sequence within conserved motif IV and other downstream motifs in its catalytic activity, truncation constructs of GALT8 at amino acid residue G_293_ within the GxxYxxS motif (GALT8 CΔ106) and I_230_ just before the DDD motif (GALT8 CΔ168), were generated (Figure 5A). GALT8 CΔ106 therefore mimics the T-DNA insertion in the *galt8* mutant line. The conserved motif GxxYxxS of GT31 enzymes is part of a flexible G-loop, where the conserved glycine residue is involved in donor binding (Taujale et al., 2020) and is located near the active site (Petit et al., 2020). The DXD motif, in this case, D_231_D_232_D_233_, was mutated to N_231_D_232_N_233_ (GALT8 NDN) (Figure 5A) to generate a catalytic knockout construct.

These constructs were individually expressed in tobacco leaves and their protein levels and enzymatic activities measured as described above. In the WT full-length GALT8 control, oligosaccharide products up to DP5 were detected (Figure 5B). However, mutation in the predicted catalytic DDD site and both truncations (at either positions I_230_ or G_293_) led to a complete loss of GalT activity as only the β-Gal-NBD acceptor could be detected (Figure 5B). The presence of GALT8 protein in the MM was confirmed by Western blot in all four samples using an anti-GALT8 antibody (Figure 5C), indicating the lack of detectable enzyme activity was not due to an absence of GALT8 variant protein. WT GALT8, GALT8 NDN, GALT8 CΔ168, and GALT8 CΔ106 have predicted MWs of 45.2, 45.2, 26.3, and 33.3 kDa, respectively (red boxes, Figure 5C). Interestingly, bands with approximately twice the predicted MW of the GALT8 variants were observed (blue boxes, Figure 5C) and suggest the presence of homodimers. Upper bands may represent higher-order GALT8 protein complexes. Having confirmed that the GALT8 variant proteins are expressed and produce proteins of the expected sizes, we can conclude that the C-terminal domain containing the DDD and other conserved motifs is critical for the enzymatic activity of GALT8.

### Phenotypes of the *galt8* Mutant Lines

**FIGURE 6:**
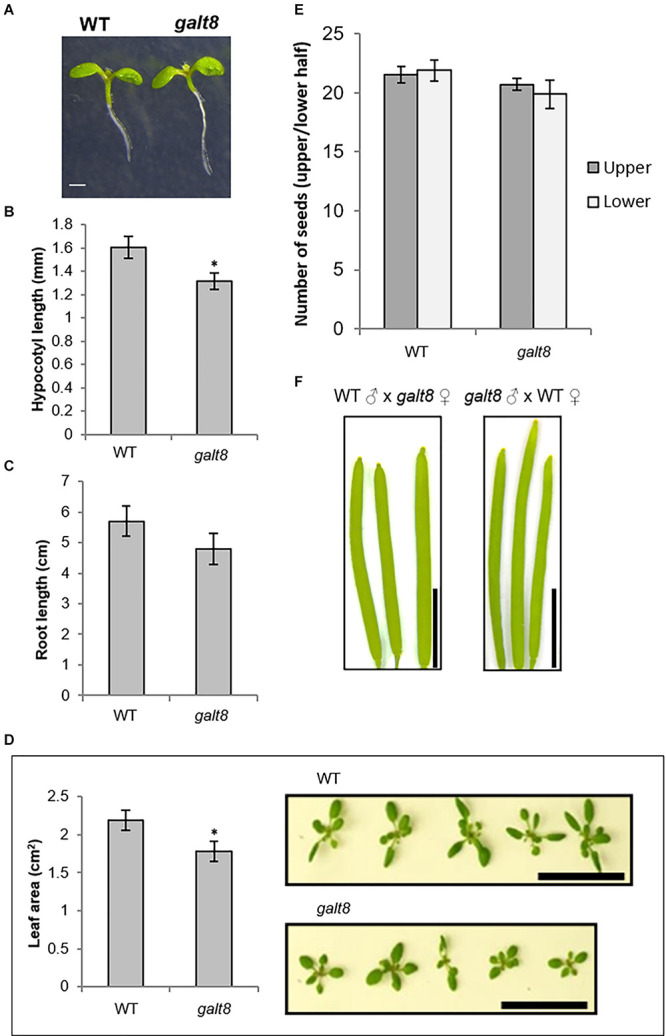
Hypocotyl length, root length, and seedling leaf area of *galt8* and WT Col-0 lines. **(A)** Light-grown seedlings (6 days) of WT Col-0 and *galt8.* **(B)** Bar graph of the average hypocotyl length of 6 days old light-grown *galt8* and WT Col-0 seedlings (*n* = 10). Asterisk indicates statistical significance (Student’s *T*-test, *p* < 0.05). **(C)** Bar graph of the average root length of *galt8* and WT Col-0 seedlings 14 days post-germination (*n* = 10) (Student’s *T*-test, p > 0.05). **(D)** Average seedling leaf area of *galt8* mutants compared to WT Col-0 two weeks after germination (*n* = 15; one-way Student’s *T*-test, *p* < 0.05). Overhead view of WT Col-0 (upper) and *galt8* (lower) seedlings two weeks after germination to show relative leaf area. **(E)** Average number of seeds of *galt8* mutants compared to WT Col-0 (*n* = 15 individuals; two-tail Student’s *T*-test, Upper p = 0.39, Lower p = 0.21). **(F),** Siliques from reciprocal crosses between *galt8* and WT Col-0. Error bars indicate SE. Scale bar = 1 mm **(A)**; Scale bar = 5 cm **(D)**; Scale bar = 0.5 cm **(F)**.

**FIGURE 7:**
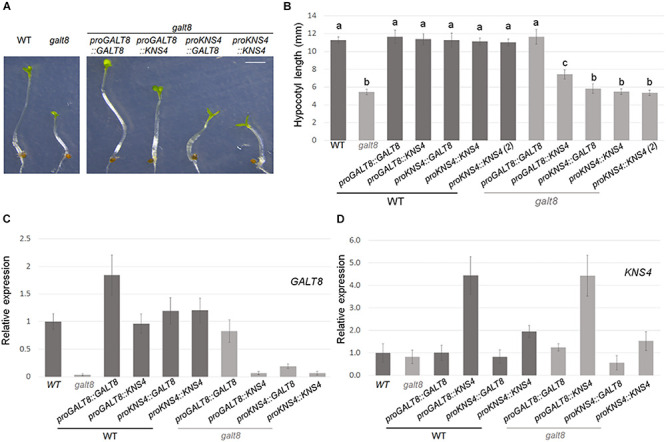
Expression of *GALT8* or *KNS4/UPEX1* in *Arabidopsis* WT or *galt8* mutant lines. **(A)**, Hypocotyl phenotype of 4 days dark-grown seedlings of *Arabidopsis* WT Col-0, *galt8*, WT overexpressing (OE) lines and *galt8* complementation lines. **(B)**, Mean hypocotyl length ± standard deviation (SD) of the same lines. Values with different letters (a,b,c) represent a statistically significant difference (Student *T*-test, *p* < 0.01). **(C,D)**, Quantitative gene expression analysis of *GALT8* and *KNS4/UPEX1*, respectively, in 6 days light-grown seedlings. Mean expression ± SD of two biological replicates is shown relative to WT. Scale bar = 2 mm **(A)**.

Seed germination and seedling establishment phases are potentially affected by defects in micropylar endosperm development, hence the germination rate and seedling morphology of *galt8* mutants were analyzed to examine the effect of inactive GALT8. No difference in germination rate of *galt8* mutant seeds was observed relative to WT (data not shown). However, a noticeably shorter hypocotyl was observed in *galt8* seedlings compared to WT when grown in either the light or dark (Figures 6A, 7A, respectively). The average hypocotyl length of 6-day-old light-grown *galt8* seedlings (1.3 ± 0.1 mm) was significantly shorter compared with those of WT Col-0 (1.6 ± 0.1 mm) (Student’s *T*-test, *p* < 0.05) (Figure 6B). A significant reduction in hypocotyl length was also observed in 4 days old dark-grown *galt8* (5.5 ± 0.3 mm) versus WT (11.3 mm ± 0.3mm) seedlings (Student’s *T*-test, *p* < 0.01). WT Col-0 and *galt8* roots of light-grown seedlings did not show a significant difference in root length (5.7 ± 0.5 cm and 4.8 ± 0.5 cm, respectively) (Figure 6C). In contrast, the average seedling leaf area of *galt8* (1.78 ± 0.13 cm^2^) mutants was significantly smaller than that of WT Col-0 plants (2.19 ± 0.13 cm^2^) (Student’s *T*-test, *p* < 0.05) (Figure 6D), demonstrating that there are developmental deficiencies in *galt8* mutant seedlings. Together, these phenotypes indicate that the *GALT8* mutation impacts seedling establishment in *Arabidopsis*. By maturity, no significant differences were observed in stem height between WT Col-0 (32.5 ± 0.9 cm, *n* = 8) and *galt8* (32.1 ± 0.7 cm, *n* = 8) plants.

Siliques from *galt8* and WT Col-0 plants were examined to reveal whether any defects in seed development could be observed. A similar number of seeds were produced both in the upper and lower half of *galt8* and Col-0 siliques (Figure 6E). Additionally, siliques from reciprocal crosses between *galt8* and WT Col-0 plants exhibited similar lengths (Figure 6F), suggesting the absence of a pollen defect in *galt8* mutants. To confirm this, the morphology of *galt8* and WT Col-0 pollen was analyzed by light microscopy and scanning electron microscopy (SEM) (Supplementary Figures 5A,B, respectively). These analyses revealed that *galt8* mutants produce pollen grains that share similar surface morphology and overall phenotype with that of WT Col-0, suggesting that any phenotypic defects in *galt8* are unlikely a result of an impairment in male gametophyte development.

### Genetic Complementation of *galt8*

In order to determine if GALT8 and KNS4/UPEX1, both sub-clade II members of GT31 Clade 10 with β-(1,3)-GalT activity, are functionally equivalent, genetic complementation experiments were performed. Constructs with each of the combinations of the *GALT8* or *KNS4/UPEX1* promoter with either the *GALT8* or *KNS4/UPEX1* coding region were generated and transformed into *galt8* mutants as well as WT Col-0 plants. Hypocotyl lengths of plants homozygous for one of each of the five complementation constructs were analyzed after 4 days growth on MS medium in the dark rather than the light for greater ease of phenotype quantification. *Galt8* mutants homozygous for the *proGALT8:GALT8* construct showed full complementation of the hypocotyl length phenotype. The *GALT8* promoter driving *KNS4/UPEX1* (*proGALT8*:*KNS4/UPEX1*) resulted in partial complementation with *galt8* complementation lines displaying a significant increase in hypocotyl length compared to *galt8*, but not to the level of WT (Student’s *T*-test, *p* < 0.01) (Figures 7A,B and Supplementary Figure 6A). Expression of either *GALT8* or *KNS4/UPEX1* driven by the *KNS4/UPEX1* promoter [both the longer (2006 bp) and shorter (1327 bp) variants (see section “Methods”)] in *galt8* mutants resulted in no significant change in hypocotyl length compared to *galt8.* The presence in homozygous state of any of the five complementation cassettes in the WT Col-0 background appeared to have no effect on hypocotyl length (Figure 7B).

Quantitative PCR analyses of hypocotyls of 6 day-old light-grown seedlings of WT, *galt8*, and *galt8*-complementation lines showed that the *GALT8* promoter restored the expression of *GALT8* in *galt8*-complementation lines to WT levels (Figure 7C and Supplementary Figure 6B). In contrast, when *KNS4/UPEX1* expression was driven by the *GALT8* promoter in *galt8* complementation lines, significantly higher transcript levels were observed compared to WT (Figure 7D and Supplementary Figure 6C). Therefore, an insufficient level of *KNS4/UPEX1* transcript was not the reason for the hypocotyl phenotype not being restored in these lines. On the other hand, the *KNS4/UPEX1* promoter only partly restored the expression level of *GALT8* in *galt8*-complemented mutants, indicating that this promoter is weaker in comparison to the *GALT8* promoter (Figure 7C and Supplementary Figure 6B). The observation of reduced levels of *KNS4/UPEX1* expression in proKNS4:KNS4 lines compared to proGALT8:KNS4 lines also supports this interpretation (Figure 7D and Supplementary Figure 6C). Together, these data suggest that *GALT8* has some but not complete functional overlap with *KNS4/UPEX1.*

## Discussion

To test the hypothesis that GALT8 has a similar biochemical activity to KNS4/UPEX1, GALT8 catalytic specificity was first examined using different UDP-sugar donors as substrates in an *in vitro* enzyme assay using a synthetic Gal-NBD acceptor. GALT8 was shown to have activity only when UDP-Gal but not UDP-Glc, UDP-Ara*p* or UDP-Xyl was used as a donor thereby confirming the bioinformatics predictions that GALT8 is a GalT. Using a multi-pronged structural approach including RP-HPLC of enzyme assay products, linkage-specific enzymatic digestion and MS-based analyses, GALT8 was shown to be a β-(1,3)-GalT with essentially similar biochemical activity as KNS4/UPEX1 (Suzuki et al., 2017). β-Glc Yariv binding confirmed the enzyme products were precursors of type II AG glycans typically found attached to AGP protein backbones, although such glycans have been observed in pectin extracts (Immerzeel et al., 2006; Tan et al., 2013) and references therein. Our current data do not allow us to distinguish between these possibilities and hence we are unable to conclude whether GALT8 plays a role in either AGP or pectin biosynthesis or both.

To confirm that Gal was the incorporated monosaccharide into *in vitro* assay products derived from GALT8 activity, we tested the susceptibility of these products to enzymatic digestion. *E. coli* β-galactosidase digestion of the GALT8 reaction products resulted in the breakdown of all products and the β-Gal-NBD acceptor to the free NBD tag (Figure 1C). This confirmed that the products made by GALT8 were β-D-Gal oligosaccharides. In terms of the linkage type, a type II AG-specific hydrolase, SGalase1, and an endo-β-(1,6)-galactanase, *Nc*6GAL, were used to digest the galacto-oligosaccharide products. SGalase1 hydrolyzed the GALT8 products to β-Gal-NBD, whilst *Nc*6GAL was not able to digest the galacto-oligosaccharide products (Figure 1D), indicating the absence of β-(1,6)-Gal branches. This confirms that the major products formed by GALT8 were an oligomeric β-(1,3)-D-galacto-oligosaccharide series of DP 2-5. ESI-MS and -MS^n^ of the individual peaks obtained from the biochemical enzyme assay further supported the results of the RP-HPLC and the enzymatic digestions. The positive reactivity to β-Glc Yariv dye of the higher DP galacto-oligosaccharides from the GALT8 enzyme assay indicates that the products of the GALT8 enzyme assay are β-(1,3)-galacto-oligosaccharides, since β-Glc Yariv binds preferentially to an unsubstituted β-(1,3)-Gal backbone with DP > 5 (Kitazawa et al., 2013; Paulsen et al., 2014).

This β-(1,3)-GalT activity is consistent with the predictions from the phylogenetic tree shown in Supplementary Figure 1. Clade 10, in which GALT8 and KNS4/UPEX1 belong, is divided into three robust sub-clades, III, I + II, and IV (Qu et al., 2008) with very high bootstrap support. Members of each sub-clade are predicted to have similar rather than distinct biochemical activities. Clade III contains the three demonstrated Hyp*-O-*GalTs HPGT1-3, consistent with their similar enzymatic activities (Ogawa-Ohnishi and Matsubayashi, 2015). There appears to be a core set of sequences within sub-clade I (At2g32430, At1g05170, At4g26940, and At1g11730) and sub-clade II (GALT8, At1g77810, and KNS4/UPEX1), the latter all displaying β-(1,3)-GalT activity catalyzing either single or multiple Gal additions. However, the sub-division of sub-clades I and II is not entirely clear. The consistent positioning of GALT31A at the base of sub-clades I and II suggests that this GT31 member may be part of a separate sub-clade. *At*GALT31A was previously reported to have β-(1,6)-GalT activity (Geshi et al., 2013). However, we have been unable to replicate the β-(1,6)-GalT activity of AtGALT31A, rather we have shown using the same biochemical assay described in this study that it has β-(1,3)-GalT activity (Zeng et al., unpublished data). Another β-(1,6)-GalT, *At*GALT29A (At1g08280), which elongates β-(1,6)-galactan side chains to form β-(1,6)-Gal branches on the β-(1,3)-galactan backbone of type II AGs, belongs to CAZy Family GT29 (Dilokpimol et al., 2014). It is possible that other GTs are responsible for synthesizing β-(1,6)-Gal linkages in *Arabidopsis thaliana*. The function of a sub-clade I and IV member has yet to be reported, although it seems likely that these enzymes would have similar biochemical activities to other Clade 10 members (Supplementary Figure 1).

*Galt8* mutant lines were observed to have seedling phenotypes, notably, poor seedling establishment and reduced rosette leaf area (Figures 6A-D). *GALT8* is expressed in the central cell and synergids before pollination and then afterward, in the micropylar endosperm, which provides a plausible explanation for its seedling phenotypes. These phenotypes suggest that not any β-(1,3)-GalTs is able to compensate for the loss of *GALT8* in seedlings. In comparison, *KNS4/UPEX1* is specifically expressed in the male gametophyte and *kns4/upex1* mutants display a pollen-defective phenotype (Dobritsa et al., 2011; Li et al., 2017; Suzuki et al., 2017). Although *GALT8* is also expressed in anthers (Figure 4C), *galt8* mutants did not exhibit any observable pollen phenotype (Figures 6E,F and Supplementary Figure 5), suggesting that other genes may have functional overlap with *GALT8* in pollen. Given its expression pattern, it is plausible that either *KNS4/UPEX1* expression in the anther could provide sufficient GalT activity under normal conditions to negate a pollen phenotype in *galt8* mutants or a number of other GT31 Clade 10 (subclades I and IV) members that are also expressed in this tissue could potentially fulfill this role (Arabidopsis eFP Browser^4^). Another possibility is that GALT8 has a divergent function to KNS4/UPEX1 in the anther that might manifest into a noticeable pollen phenotype in *galt8* mutants when exposed to a/biotic stress. Further work is required to reveal which GT31 member/s can substitute the role/s of GALT8 during pollen development.

The observation of phenotypes in both *kns4/upex1* and *galt8* mutant lines suggests impairment of β-(1,3)-GalTs involved in type II AG backbone biosynthesis may lead to stronger phenotypes than other enzymes that participate in terminal sugar addition to type II AG moieties. Examples of enzymes that are AGP-specific and exhibit weak phenotypes in mutant *Arabidopsis* plants are the fucosyltransferases FUT4 and FUT6. Mutant *fut4*, *fut6*, and *fut4/fut6* plants showed no phenotypic difference compared to WT Col-0 under physiological environments, but showed reduced root growth under elevated NaCl conditions (Wu et al., 2010; Liang et al., 2013; Tryfona et al., 2014; Soto et al., 2021). Another group of GTs participating in AGP glycan biosynthesis is the β-glucuronosyltransferases (GlcATs; GT14) (Ajayi and Showalter, 2020). Phenotypic analyses of single *glcAT* mutants revealed only mild phenotypes, with double and triple mutant combinations required for more phenotypic enhancement (Knoch et al., 2013; Lopez-Hernandez et al., 2020; Zhang et al., 2020). A potential reason why phenotypes resulting from mutations in β-(1,3)-GalT genes involved in generating the AG backbone may be more severe is that either more AGP backbones are affected in such instances and/or a greater proportion of total AG glycan moiety function is affected. Further work is needed to differentiate between such possibilities.

Online microarray data, previously published promoter-reporter findings, and seedling defects show that *GALT8* affects the female gametophyte and later, micropylar endosperm development. *KNS4/UPEX1*, on the other hand, is specifically expressed in the tapetal cells of young anthers and affects pollen exine development (Dobritsa et al., 2011; Li et al., 2017; Suzuki et al., 2017). Based on the enzyme assays of GALT8 and KNS4/UPEX1, the two GTs share similar biochemical functions as β-(1,3)-GalTs but in distinct tissues. To determine whether *GALT8* and *KNS4/UPEX1* can functionally complement each other, a *galt8*-complementation experiment was designed such that the native *GALT8* promoter was used to drive expression of either CDS to eliminate any spatio-temporal differences in their expression. As expected, the *GALT8* promoter restored both the expression of *GALT8* in *galt8* complementation lines to WT levels, as well as the WT phenotype (Figure 7 and Supplementary Figure 6), indicating that the 852 bp promoter contains all the sequence features for proper expression. In contrast, when *KNS4/UPEX1* expression was driven by the *GALT8* promoter in *galt8* complementation lines, significantly higher transcript levels relative to WT were observed but the hypocotyl phenotype was only partially restored. This suggests that GALT8 and KNS4/UPEX1 have only partly overlapping function, that is, there is functional divergence between these GT31 members to cause only partial and not full complementation.

An example of partial functional conservation in another plant GT is the IRX10 orthologs of *Physcomitrella patens* and *A. thaliana*. Despite the sequence similarity (∼76%) between the IRX10 orthologs in these two species, the *Physcomitrella IRX10* gene is only able to partially rescue the *Arabidopsis irx10/irx10-L* double mutant, indicating that there has been neo- or sub-functionalization during their evolution (Hörnblad et al., 2013). Although, the high sequence identity of IRX10 orthologs in *Arabidopsis* and *Physcomitrella* did not result in full functional complementation, partially overlapping functions between the two proteins can still be explained by their sequence conservation. In the case of GALT8 and KNS4/UPEX1, the percentage identity of their full-length protein sequences is around 55%; however, the sequences surrounding the DDD motif involved in catalysis and the conserved motifs IV – VI further downstream in the C-terminal domain are more highly conserved (Figure 4B). Thus, it is possible that they share similar enzymatic activity but are functionally distinct, for example, through their differential interactions with protein partners and/or ligands. Another possibility that could explain the partial but not full complementation of the *galt8* mutant by KNS4/UPEX1 is that this enzyme may be involved in the biosynthesis of a set of type II AG chains, either on AGPs and/or pectins, most likely the sidechains of RG I, that does not completely overlap with those made by GALT8. Comparison of the molecular interactions of GALT8 and KNS4/UPEX1 together with a detailed analysis of the pectin and AGP components of the WT, mutant and complementation lines is needed to shed further light on the functional specialization of these GT31 enzymes.

Genes often exhibit a dosage effect in which the amount of the protein or enzyme product is closely correlated with the number of gene copies present (Coate et al., 2016). This means that transcript abundance must increase with gene dosage in order to increase protein abundance. Using *Arabidopsis IRX10* and *IRX10-L* as an example, it was shown that these two genes exhibit partial complementation effects and transgene dosage effects (Wu et al., 2009). When the *IRX10-L* was expressed as a single copy in the T_1_ generation and driven by the *IRX10-L* promoter, it partially complemented the *irx10/irx10-L* double mutant. When *IRX10-L* was expressed in the homozygous T_2_ generation and driven by the *IRX10-L* promoter, the wild-type appearance was restored in the *irx10/irx10−L* double mutant background (Wu et al., 2009). A reasonable interpretation of this result is that IRX10 and IRX10-L normally function in separate partially redundant biosynthetic pathways. With respect to *GALT8* and *KNS4/UPEX1*, insufficient levels of *KNS4/UPEX1* transcript was not the reason for the hypocotyl phenotype not being restored in *galt8* complementation lines (Figure 7D). In the current study, only lines homozygous for the transgene were analyzed and so transcript levels driven by the *GALT8* promoter should theoretically be similar to WT when expressed in the *galt8* mutant background. This is indeed the case for GALT8. When two copies of the pro*GALT8:GALT8* expression cassette are present in *galt8* complementation lines, transcript levels are observed to be similar to WT levels and when present in the WT background, are approximately double (Figure 7C and Supplementary Figure 6B). While the *KNS4/UPEX1* promoter was able to restore *KNS4/UPEX1* expression to WT levels, significantly more transcript, ∼4-fold higher than WT, was observed when expression was driven by the *GALT8* promoter (Figure 7D and Supplementary Figure 6C), without full phenotypic rescue. This suggests that gene dosage is not the major reason why *KNS4/UPEX1* is unable to complement the *galt8* mutant. We speculate that due to their sequence variance, GALT8 and KNS4/UPEX1 may have a different suite of protein interaction partners, possibly within a type II AG biosynthetic complex, that is responsible for their functional divergence. That GALT8 was identified on immunoblots of denaturing protein gels at molecular masses greater than monomer size (Figure 5C) is suggestive that it is part of a protein complex. Further experiments are currently underway to test this hypothesis.

Our study has shown that GALT8 is a GT31 β-(1,3)-GalT involved in type II AG synthesis of AGPs and/or pectins, with *galt8* mutants exhibiting developmental deficiencies at seedling stage. From the *galt8*-complementation experiments, it can be deduced that *GALT8* has partial functional overlap with the previously reported *KNS4/UPEX1*, another β-(1,3)-GalT in the GT31 family. Gradually, the biochemical activities of the plant GT31 family members are being elucidated, although more studies are required to determine whether the β-(1,3)-GalTs, β-(1,6)-GalTs, and possibly other enzymes that add sugars to type II AG sidechains, form biosynthetic protein complexesas is the case during the production of other plant cell wall polysaccharides. In addition, the question of whether the type II β-(1,6)-GalT activities reside either within the GT31 family or elsewhere remains to be answered. Revealing the identities of all proteins involved in the biosynthesis of type II glycans is an important first step to synthesizing a type II AG *in vitro* and to manipulating them in a specific manner *in planta* to both further dissect their functional roles and biotechnological utility.

## Data Availability Statement

The raw data supporting the conclusions of this article will be made available by the authors, without undue reservation.

## Author Contributions

JON, WZ, KF, EL, JH, AvdM, and IA conducted the experiments. JON, WZ, KF, EL, JH, MD, and AB designed the experiments. JON, WZ, KF, EL, JH, IA, AB, and MD wrote the manuscript. All authors contributed to the article and approved the submitted version.

## Conflict of Interest

The authors declare that the research was conducted in the absence of any commercial or financial relationships that could be construed as a potential conflict of interest.

## Footnotes

1 www.cazy.org/

2 http://signal.salk.edu/

3 http://bar.utoronto.ca/efp2/Arabidopsis/Arabidopsis_eFPBrowser2.html

4 http://bar.utoronto.ca/efp/cgi-bin/efpWeb.cgi
