# Supplementary material for: Biochemical and Functional Characterization of GALT8, an Arabidopsis GT31 β-(1,3)-Galactosyltransferase That Influences Seedling Development
Source: Front Plant Sci. 2021 May 25;12:678564. doi: 10.3389/fpls.2021.678564 (PMC8186459; doi:10.3389/fpls.2021.678564)
Supplement: Supplementary file 1 [file Presentation_1.pdf]

Supplementary Table 1. Primer sequences used for plant genotyping, (q)RT-PCR analysis and generating the GALT8 construct variants for heterologous expression in *Nicotiana benthamiana*.

Primer Name	Sequence (5' - 3')
Genotyping primers:	
LP(8)	GGTCTTCCTCAGCGTCTTTTC
RP(8)	AACTCGTTGCAGGATCATGTC
LBb1.3	ATTTTGCCGATTTCGGAAC
GALT8 RT-PCR primers:	
RTPCR(8)-1F	CACAAGACAATGATGTAATGGAAG
RTPCR(8)-1R	CAGCCTAAAGAAATCGTTGTATTG
RTPCR(8)-2F	CTCGGGTCATGGTTTATCGG
RTPCR(8)-2R	CGTCCCACACAGCTTTACTA
ARP9F	GAGTTCTTCACGCGATACCTCCA
ARP9R	GACCACCTTTATTAACCCCATTTACCA
GAPA1F	TGGTTGATCTCGTTGTGCAGGTCTC
GAPA1R	GTCAGCCAAGTCAACAACTCTCTG
TUA2F	ATGTGGGTGAGGGTATGGAA
TUA2R	CCGACAACCTTCTTAGTACTCCTCT
qRT-PCR primers:	
GALT8qF	ACACAGTTCAACGCCAAACA
GALT8qR	CCCACTTTGCAACTGCACTA
KNS4qF	GAGAACTCACCAAGCAGTAA
KNS4qR	TTTCTGCAGACGGCTCTGGTTTTTC
GAPdHqF	TGGTTGATCTCGTTGTGCAGGTCTC
GAPdHqR	GTCAGCCAAGTCAACAACTCTCTG
GALT8 heterologous expression construct primers:	
GALT8(1)-F	TCGAGGAATTCGGTACCATGAAGCACAACAACAAAGTCTCCAAGAGAC
GALT8(2)-R	AAGCAGGACTCTAGAGGATCCTCAAGAGAGTTTTAAATTTGCGTCCCACAC
GALT8(3)-F	TCGAGGAATTCGGTACCATGAAGCACAACAACAAAGTCTCC
GALT8(4)-R	AAGCAGGACTCTAGAGGATCCTCACGTAGCATGTCGGAAATATTTGTTA
GALT8(5)-R	AAGCAGGACTCTAGAGGATCCTCAAATCTTGACATAAAACTCAGCATCC
GALT8(6)-R	GAACTTCATCTTCAATCTTGACATAAAACTCAGCATCC
GALT8(7)-F	GATTGAAGATGAAGTTCACGTCAATCTTGGCACG
GALT8(8)-R	AAGCAGGACTCTAGAGGATCCCTAAGAGAGTTTTAAATTTGCGTCCC

Supplementary Table 2. Primer sequences used to generate constructs for *galt8* genetic complementation studies. Italicised and underlined bases indicate the restriction sites.

**Primer Name**	**Sequence (5' - 3')**
GALT8PromF_EcoRI	GATA*<u>GAATTC</u>*GGAATTTGGAAGGGTTACCG
GALT8PromR_SalI	ACGT*<u>GTCGAC</u>*TGTGAGCACACAAAGAGAGACG
GALT8F_SalI	AAGA*<u>GTCGAC</u>*ATGAAGCACAACAACAAAG
GALT8R_NcoI	ACGA*<u>CCATGG</u>*CTAAGAGAGTTTTAAATTTG
KNS4PromF_EcoRI	CATT*<u>GAATTC</u>*TCGCAAACCGGTATTACTGC
KNS4PromR_SalI	ACGT*<u>GTCGAC</u>*CTCCGCGCCTCCTTAGTG
KNS4F_SalI	AAGA*<u>GTCGAC</u>*ATGAGGGCGAAAGCTGCTTCG
KNS4R_NcoI	ACAT*<u>CCATGG</u>*TCAGACGAAGAAGCGAAAATTAG

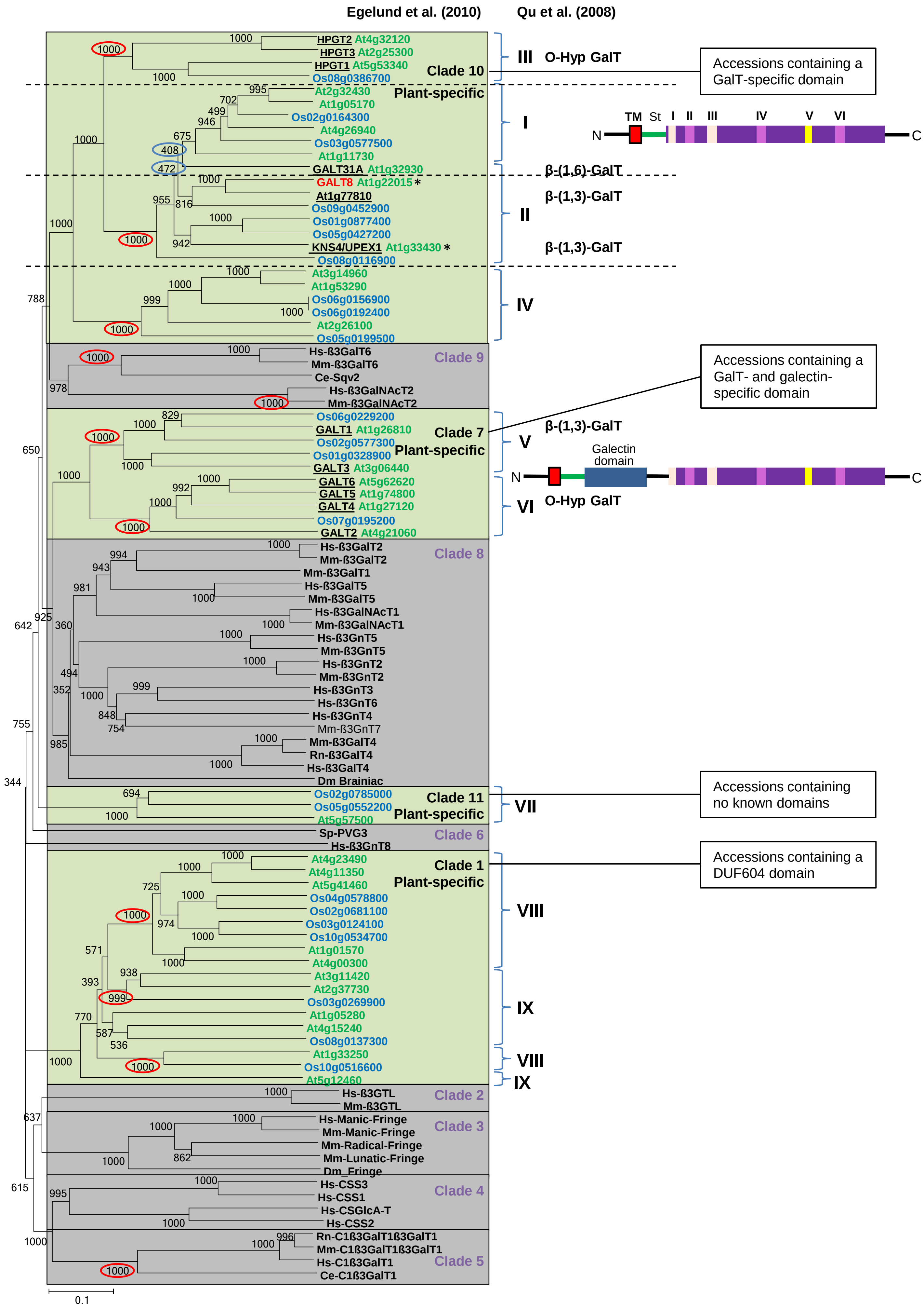
Egelund et al. (2010)
Qu et al. (2008)
HPGT2 At4g32120
HPGT3 At2g25300
HPGT1 At5g53340
Os08g0386700
Clade 10
III
O-Hyp GalT
Accessions containing a GalT-specific domain
At2g32430
At1g05170
Os02g0164300
At4g26940
Os03g0577500
At1g11730
Plant-specific
I
TM
St
N
C
GALT31A At1g32930
β-(1,6)-GalT
GALT8 At1g22015 *
At1g77810
Os09g0452900
Os01g0877400
Os05g0427200
KNS4/UPEX1 At1g33430 *
Os08g0116900
β-(1,3)-GalT
II
β-(1,3)-GalT
At3g14960
At1g53290
Os06g0156900
Os06g0192400
At2g26100
Os05g0199500
IV
Hs-ß3GalT6
Mm-ß3GalT6
Ce-Sqv2
Hs-ß3GalNAcT2
Mm-ß3GalNAcT2
Clade 9
Accessions containing a GalT- and galectin-specific domain
Os06g0229200
GALT1 At1g26810
Os02g0577300
Os01g0328900
GALT3 At3g06440
GALT6 At5g62620
GALT5 At1g74800
GALT4 At1g27120
Os07g0195200
GALT2 At4g21060
Clade 7
Plant-specific
β-(1,3)-GalT
V
Galectin domain
VI
O-Hyp GalT
Hs-ß3GalT2
Mm-ß3GalT2
Mm-ß3GalT1
Hs-ß3GalT5
Mm-ß3GalT5
Hs-ß3GalNAcT1
Mm-ß3GalNAcT1
Hs-ß3GnT5
Mm-ß3GnT5
Hs-ß3GnT2
Mm-ß3GnT2
Hs-ß3GnT3
Hs-ß3GnT6
Hs-ß3GnT4
Mm-ß3GnT7
Mm-ß3GalT4
Rn-ß3GalT4
Hs-ß3GalT4
Dm Brainiac
Clade 8
Os02g0785000
Os05g0552200
At5g57500
Clade 11
Plant-specific
VII
Accessions containing no known domains
Sp-PVG3
Hs-ß3GnT8
Clade 6
At4g23490
At4g11350
At5g41460
Os04g0578800
Os02g0681100
Os03g0124100
Os10g0534700
At1g01570
At4g00300
At3g11420
At2g37730
Os03g0269900
At1g05280
At4g15240
Os08g0137300
At1g33250
Os10g0516600
At5g12460
Clade 1
Plant-specific
Accessions containing a DUF604 domain
VIII
IX
VIII
IX
Hs-ß3GTL
Mm-ß3GTL
Clade 2
Hs-Manic-Fringe
Mm-Manic-Fringe
Mm-Radical-Fringe
Mm-Lunatic-Fringe
Dm_Fringe
Clade 3
Hs-CSS3
Hs-CSS1
Hs-CSGlcA-T
Hs-CSS2
Clade 4
Rn-C1ß3GalT1ß3GalT1
Mm-C1ß3GalT1ß3GalT1
Hs-C1ß3GalT1
Ce-C1ß3GalT1
Clade 5
0.1

Supplementary Fig. 1. Phylogenetic tree of the CAZy GT31 family.

Protein sequences from **Table 7.1** of Egelund et al. (2010) were gathered from UniProt (http://www.uniprot.org/) and aligned by ClustalX2 (http://www.clustal.org/clustal2/) using the Multiple Alignment Mode with default parameters. The phylogenetic tree was bootstrapped using 1000 replicates and drawn using MEGA (Molecular Evolutionary Genetics Analysis) software, version 5.1 (http://www.megasoftware.net/). Clades 1, 7, 10 and 11 represent plant-specific clades of the GT31 family (Egelund et al. 2010). The names given to GT31 Clades 7 and 10 members that have been characterised are shown in underlined bold black text and their activities indicated. The Clade 10 member characterised in this study is in bold red text. The clades on the right-hand side of the tree marked with blue brackets are those defined by Qu et al. (2008). Black dashed line indicate the Clade 10 sub-clades as revised in this study. Bootstrap values >700 of 1000 replicates are marked with a red ellipse, <700 that impact on sub-clade delineation are indicated with a blue ellipse. Species names are abbreviated as follows: At, *Arabidopsis thaliana* (in green); Ce, *Caenorhabditis elegans*; Dm, *Drosophila melanogaster*; Hs, *Homo sapiens*; Mm, *Mus musculus*; Os, *Oryza sativa* (in blue); Rn, *Rattus norvegicus*. Schematic diagrams of the major domains and motifs in Clade 7 and 10 members are also shown. Schematic diagrams of the major domains and motifs in Clade 7 and 10 members are also shown. N, N-terminus; C, C-terminus; TM, transmembrane region; St, stem region; purple box, PFAM GalT (PF01762) domain; small pink and yellow boxes, six major conserved motifs I-VI; blue box, galectin domain.

(A)

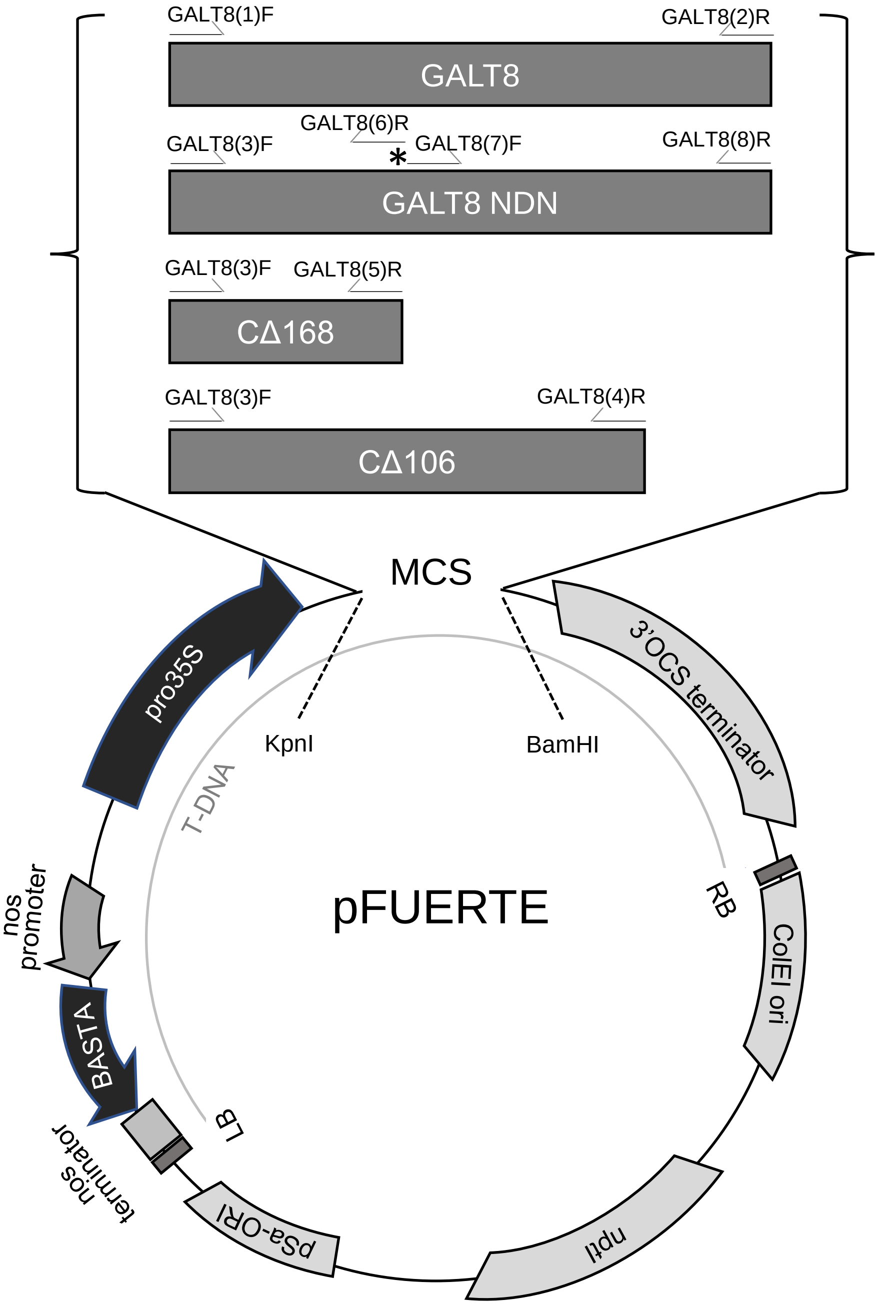
GALT8(1)F
GALT8(2)R
GALT8
GALT8(6)R
GALT8(3)F
GALT8(7)F
GALT8(8)R
*
GALT8 NDN
GALT8(3)F
GALT8(5)R
CΔ168
GALT8(3)F
GALT8(4)R
CΔ106
MCS
pro35S
KpnI
BamHI
3'OCS terminator
T-DNA
pFUERTE
RB
ColEI ori
nos promoter
BASTA
LB
nos terminator
pSa-ORI
nptI

(B)

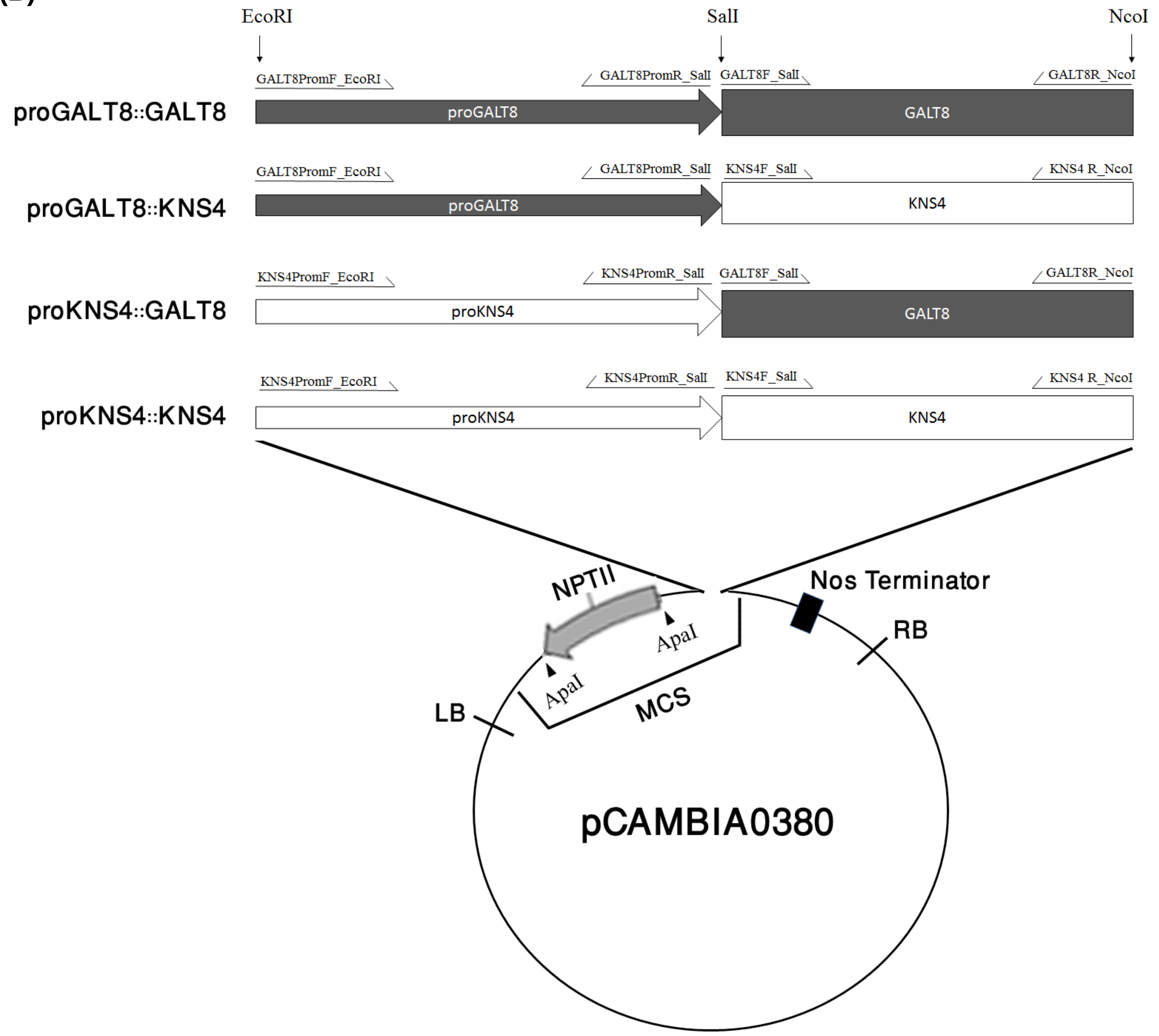

Supplementary Fig. 2. Schematic diagrams of GALT8 expression constructs (A) and *galt8* complementation constructs (B).

Cloning strategy of *GALT8* for expression in tobacco or *galt8* mutant complementation or over-expression in Arabidopsis wild type (Col-0) is outlined in Section 2.2. Cloning primers are provided in Supplemental Tables 1 (**A**) and 2 (**B**), respectively.

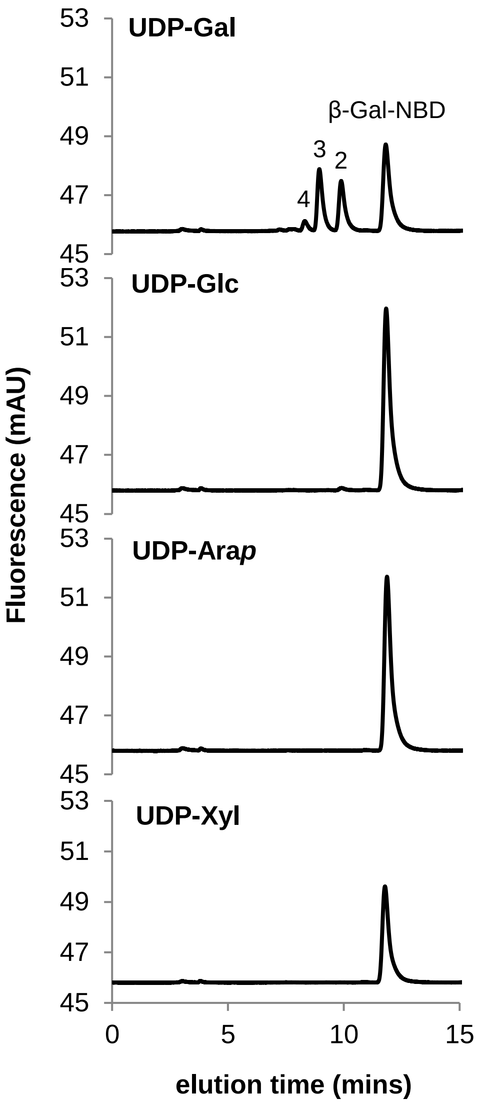

Supplementary Fig. 3. GalT activity in MMs prepared from *N. benthamiana* leaves expressing GALT8 using different nucleotide sugar donors (UDP-Gal; UDP-Glc; UDP-Ara*p*; and UDP-Xyl).

Peaks corresponding to β-$Gal_{2\text{-}4}$-NBD are labelled 2 to 4, respectively. GALT8 only utilised UDP-Gal as the sugar donor to generate products of DP2-4.

(A)

GALT8 (At1g22015)

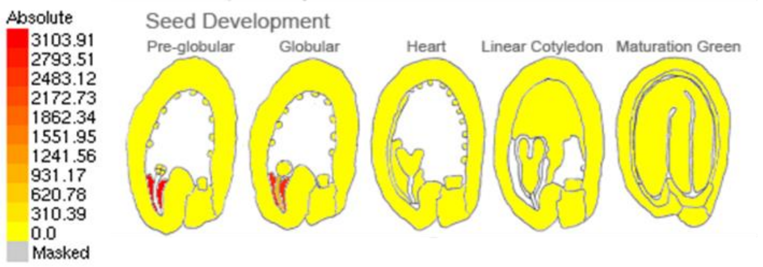

(B)

KNS4/UPEX1 (At1g33430)

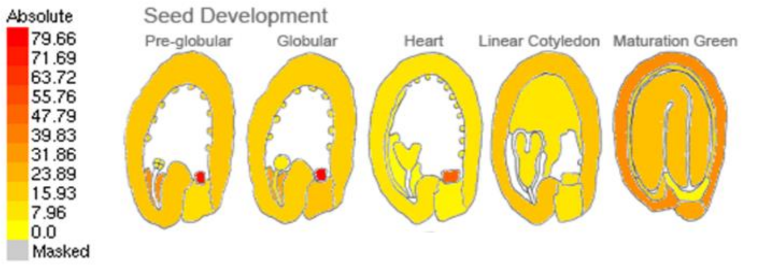

Supplementary Fig. 4. Expression profile of *GALT8* and *KNS4/UPEX1* across seed developmental stages taken from the *Arabidopsis* eFP Browser.

The heatmap on the left represents absolute transcript levels for each gene, the diagrams on the right indicate the seed tissues in which expression is observed. **(A),** *GALT8*; **(B),** *KNS4/UPEX1*. *GALT8* has significantly higher expression than *KNS4/UPEX1* in the seed, specifically in the micropylar endosperm in the pre-globular and globular stages.

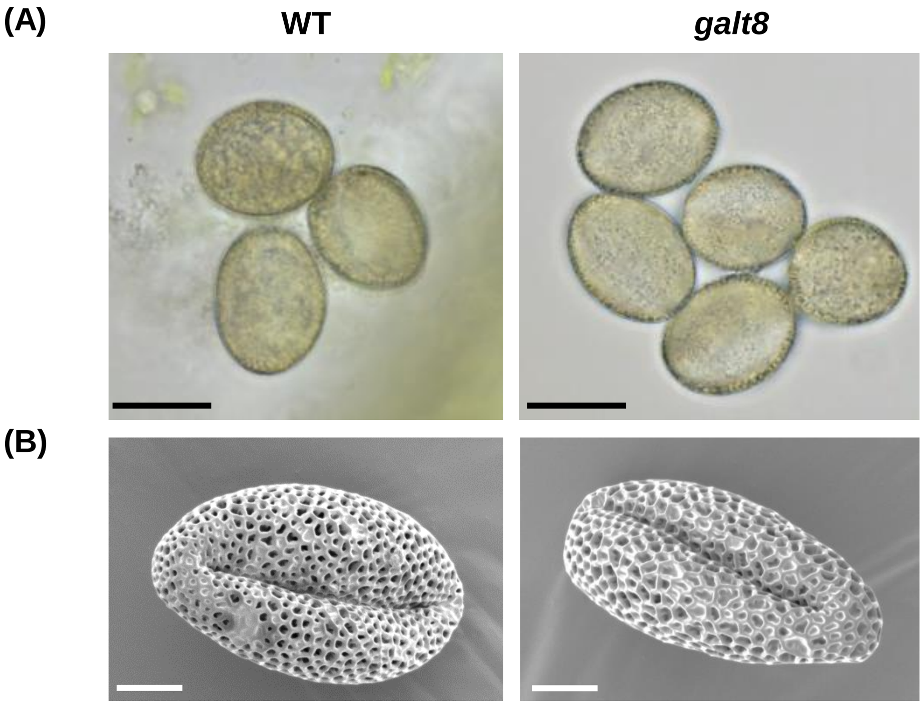

Supplementary Fig. 5. Light and scanning electron micrographs of *galt8* and WT pollen.

(**A)**, WT and *galt8* pollen as viewed by light microscopy, showing absence of visible defects in *galt8* pollen. (**B)**, Surface view of *galt8* and WT pollen grain as viewed by SEM, revealing their similar morphology. Scale bar (**A**) = 20 µm. (**B**) = 5 µm.

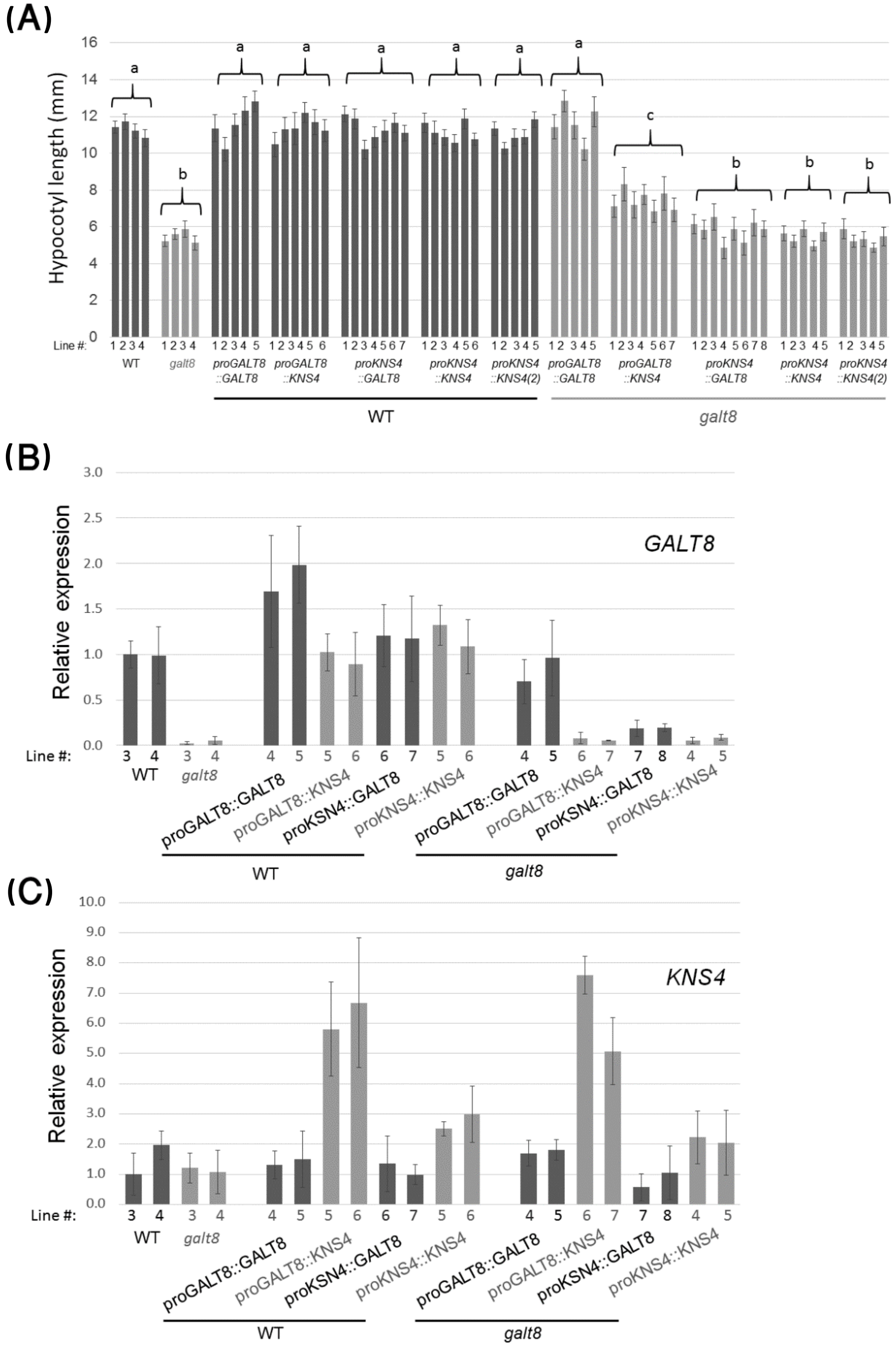

Supplementary Fig. 6. Mean hypocotyl length of *Arabidopsis* WT or *galt8* mutant lines expressing *GALT8* or *KNS4/UPEX1*.

(**A**), Mean hypocotyl length (3 technical replicates +/- SD of individual 4 days dark-grown Arabidopsis WT (Col-0), *galt8*, WT over-expressing (OE) lines and *galt8* complemented lines. Values with different letters (a,b,c) represent a statistically significant difference (Student T-test, $p<0.01$). (**B**), Quantitative gene expression analysis of *GALT8*, and (**C**), quantitative gene expression analysis of *KNS4/UPEX1* in WT Col-0, *galt8*, WT OE lines and *galt8* complemented lines. Mean expression +/- SD of 3 technical replicates is shown relative to WT.
